# Preliminary phytochemical screening and *In vitro *antioxidant activities of the aqueous extract of *Helichrysum longifolium *DC

**DOI:** 10.1186/1472-6882-10-21

**Published:** 2010-05-14

**Authors:** Olayinka A Aiyegoro, Anthony I Okoh

**Affiliations:** 1 Applied and Environmental Microbiology Research Group (AEMREG), Department of Biochemistry and Microbiology, University of Fort Hare, Private Bag X1314, Alice 5700, South Africa

## Abstract

**Background:**

Many oxidative stress related diseases are as a result of accumulation of free radicals in the body. A lot of researches are going on worldwide directed towards finding natural antioxidants of plants origins. The aims of this study were to evaluate *in vitro *antioxidant activities and to screen for phytochemical constituents of *Helichrysum longifolium *DC. [Family Asteraceae] aqueous crude extract.

**Methods:**

We assessed the antioxidant potential and phytochemical constituents of crude aqueous extract of *Helichrysum longifolium *using tests involving inhibition of superoxide anions, DPPH, H_2_O_2_, NO and ABTS. The flavonoid, proanthocyanidin and phenolic contents of the extract were also determined using standard phytochemical reaction methods.

**Results:**

Phytochemical analyses revealed the presence of tannins, flavonoids, steroids and saponins. The total phenolic content of the aqueous leaf extract was 0.499 mg gallic acid equivalent/g of extract powder. The total flavonoid and proanthocyanidin contents of the plant were 0.705 and 0.005 mg gallic acid equivalent/g of extract powder respectively. The percentage inhibition of lipid peroxide at the initial stage of oxidation showed antioxidant activity of 87% compared to those of BHT (84.6%) and gallic acid (96%). Also, the percentage inhibition of malondialdehyde by the extract showed percentage inhibition of 78% comparable to those of BHT (72.24%) and Gallic (94.82%).

**Conclusions:**

Our findings provide evidence that the crude aqueous extract of *H. longifolium *is a potential source of natural antioxidants, and this justified its uses in folkloric medicines.

## Background

Living cells may generate free radicals and other reactive oxygen species by-products as a results of physiological and biochemical processes. Free radicals can cause oxidative damage to lipids, proteins and DNA, eventually leading to many chronic diseases, such as cancer, diabetes, aging, and other degenerative diseases in humans [1]. Plants are endowed with free radical scavenging molecules, such as vitamins, terpenoids, phenolic acids, lignins, stilbenes, tannins, flavonoids, quinones, coumarins, alkaloids, amines, betalains, and other metabolites, which are rich in antioxidant activity [2,3]. Studies have shown that many of these antioxidant compounds possess anti-inflammatory, antiatherosclerotic, antitumor, antimutagenic, anticarcinogenic, antibacterial, and antiviral activities [4,5]. The ingestion of natural antioxidants has been associated with reduced risks of cancer, cardiovascular disease, diabetes, and other diseases associated with ageing, [6,7], and in recent years, there has been a worldwide trend towards the use of the natural phytochemical present in berry crops, teas, herbs, oilseeds, beans, fruits and vegetables [8-10].

The genus *Helichrysum *Mill. belongs to the Asteraceae family and consists of an estimated 600 species. The name is derived from the Greek words *helisso *(to turn around) and *chrysos *(gold). Common name include "strawflower". It occurs in Africa (with 244 species in South Africa), Madagascar, Australasia and Eurasia. The plants may be annuals, herbaceous perennials or shrubs, growing to a height of 90 cm http://en.wikipedia.org/wiki/Strawflower. *Helichrysum *species are used extensively for stress-related ailments and as dressings for wounds normally encountered in circumcision rites, bruises, cuts and sores [11,12]. Different compounds like phenolics e.g. flavonoids and chalcones, phthalides, α-pyron derivatives, terpenoids, essential oils, volatiles and fatty acids have been found in the genus [13].

*Helichrysum longifolium *leaves are used by the "Pondos" to treat circumcision wounds. The leaves are heated over very hot ash before being used as a bandage for the treatment of wounds after circumcision [11]. Among the Xhosa-speaking tribes in South Africa, circumcision is not just a surgery; it is a cultural ceremony by which men are separated from boys. Traditionally, the wound caused by circumcision is bandaged with mashed leaves of *Helichrysum pedunculatum *Hilliard & Burtt., *H*. *appendiculatum *Hilliard & Burtt. or *H. longifolium *DC (Family Asteraceae). However, traditional circumcision has a high risk of infection. Information on *H. longifolium *is scanty in available literatures thus suggesting that not much work has been done on the antioxidant potentials of this specie. *Helichrysum longifolium *is a plant that has shown potential as a source of chemotherapeutic compounds [11,14]. Phytochemical studies have revealed that the plant is rich in flavonoids and other water soluble polyphenolic compounds [12]. While the antibacterial potentials of *H. longifolium *extracts have previously been studied and reported by our research group [15,16]. This present study, therefore investigated the phytochemical compositions, the *in vitro *antioxidant and free radical scavenging potential of this plant.

## Methods

### Plant material

Leaves of *H. longifolium *were collected in December 2007 from a farm at Kidd's Beach, Eastern Cape Province of South Africa. The plant materials were compared with the voucher specimen earlier collected from the same spot and deposited at the Griffin's Herbarium of the Plant Science building of the University of Fort Hare in Alice; with herbarium number AYK/2007/HL/2. The Plant materials were later confirmed by the curator of the Herbarium to be *H. longifolium*. The leaves were picked and washed with water to remove all unwanted plant materials and sand, air dried under light exposure (27°C-30°C for 7 days), pulverized in a mill (CHRISTY LAB MILL, Christy and Norris Ltd; Process Engineers, Chelmsford, England) and stored in an airtight container for further use.

### Preparation of extract

The powdered plant material (200 g) was extracted thrice in distilled water (5.5 L; 27°C-30°C) on shaker (Stuart Scientific Orbital Shaker, UK) for 48 hours. The extract was filtered using a Buchner funnel and Whatman No.1 filter paper. The filtrate of aqueous extract obtained was quickly frozen at -40°C and dried for 48 h using a freeze dryer (Savant Refrigerated vapor Trap, RV T41404, USA) to give a yield of 30 g of dry extract. The resulting extract was reconstituted with distilled water to give desired concentrations used in this study.

### Chemicals

All chemicals were of highest purity (≥ 99.0%). Ferric chloride, HCl, Dragendorff's reagent, magnesium metal strips, methanol, gallic acid, commercial saponins were purchased from BDH, England, blood agar from Biolab, South Africa, chloroform, H_2_SO_4_, Folin-Ciocalteu reagent, Na_2_CO_3_, vanillin, aluminium chloride, potassium acetate, phosphate buffer, K_3_Fe(CN)_6_, trichloroacetic acid (TCA), 2-thiobarbituric acid (TBA), thiocyanate (FTC), butylated hydroxyl toluene (BHT), 2, 2-diphenyl-1-picrylhydrazyl (DPPH), 2, 2'-azino-bis (3-ethylbenzthiazoline-6-sulphonic acid (ABTS), potassium persulphate, sodium nitroprusside, hydrogen peroxide, sulfanilic acid, glacial acetic acid, naphthylethylenediamine dichloride, potassium metabisulphite (PMS), NADH were all purchased from Merck, USA.

### Phytochemical screening of the plant extract

A small portion of the dry extract was used for the phytochemical tests for compounds which include tannins, flavonoids, alkaloids, saponins, and steroids in accordance with the methods of [17,18] with little modifications. Exactly 1.0 g of plant extract was dissolved in10 ml of distilled water and filtered (using Whatman No 1 filter paper) A blue colouration resulting from the addition of ferric chloride reagent to the filtrate indicated the presence of tannins in the extract. Exactly 0.5 g of the plant extract was dissolved in 5 ml of 1% HCl on steam bath. A millilitre of the filtrate was treated with few drops of Dragendorff's reagent. Turbidity or precipitation was taken as indicative of the presence of alkaloid. About 0.2 g of the extract was dissolved in 2 ml of methanol and heated. A chip of magnesium metal was added to the mixture followed by the addition of a few drops of concentrated HCl. The occurrence of a red or orange colouration was indicative of the flavonoids. Freshly prepared 7% blood agar plate was used and wells were made in it. The crude extract dissolved in 10% methanol was used to fill the wells bored in the blood agar plates. Ten percent methanol was used as a negative control while commercial saponin solution was used as a positive control. The plates were incubated at 35°C for 6 h. complete haemolysis of the blood around the extract was indicative of saponin. About 0.5 g of the extract was dissolved in 3 ml of chloroform and filtered. Concentrated H_2_SO_4 _was carefully added to the filtrate to form lower layer. A reddish brown colour at the interface was taken as positive for steroid ring.

### Determination of total phenol

The amount of phenol in the aqueous leaf extract of *H. longifolium *was determined with Folin-Ciocalteu reagent using the method of Spanos [16] as modified by the Crop Research Institute Report [19]. 2.5 ml of 10% Folin-Ciocalteu reagent and 2 ml of Na_2_CO_3 _(2% w/v) was added to 0.5 ml of each sample (3 replicates) of plant extract solution (1 mg/ml). The resulting mixture was incubated at 45°C with shaking for 15 min. The absorbance of the samples was measured at 765 nm using UV/visible light. Results were expressed as milligrams of Gallic acid (0-0.5 mg/ml) dissolved in distilled water.

### Estimation of total flavonoids

Aluminum chloride colorimetric method was used for flavonoids determination. One millilitre (1 ml) of sample (1 mg/ml) was mixed with 3 ml of methanol, 0.2 ml of 10% aluminum chloride, 0.2 ml of 1 M potassium acetate and 5.6 ml of distilled water and remains at room temperature for 30 min. The absorbance of the reaction mixture was measured at 420 nm with UV visible spectrophotometer. The content was determined from extrapolation of calibration curve which was made by preparing gallic acid solution (0-0.8 mg/ml) in distilled water. The concentration of flavonoid was expressed in terms of mg/ml.

### Determination of total proanthocyanidins

Total proanthocyanidin was determined based on the procedure of Sun et al. [20]. The mixture of 3 ml of vanillin-methanol (4% v/v), 1.5 ml of hydrochloric acid was added to 0.5 ml (1 mg/ml) of aqueous extract and vortexed. The resulting mixture was allowed to stand for 15 min at room temperature followed by the measurement of the absorbance at 500 nm. Total proanthocyanidin content was expressed as gallic acid equivalent (mg/ml) from the standard curve.

### Determination of reducing power

The reducing power of the extract was evaluated according to the method of Oyaizu [21]. The mixture containing 2.5 ml of 0.2 M phosphate buffer (pH 6.6) and 2.5 ml of K_3_Fe(CN)_6 _(1%w/v) was added to 1.0 ml of the extract dissolved in distilled water. The resulting mixture was incubated at 50°C for 20 min, followed by the addition of 2.5 ml of TCA (10% w/v). The mixture was centrifuged at 3000 rpm for 10 min to collect the upper layer of the solution (2.5 ml), mixed with distilled water (2.5 ml) and 0.5 ml of FeCl_3 _(0.1%, w/v). The absorbance was then measured at 700 nm against blank sample.

### Antioxidant assay

The antioxidant activity of the aqueous plant extract was determined using ferric thiocyanate (FTC) and thiobarbituric acid (TBA) methods. The FTC method was used to measure the amount of peroxide at the beginning of peroxidation while TBA method was used to measures free radicals present after peroxide oxidation.

### Ferric thiocyanate (FTC) method

The standard method described by Kikuzaki et al. [22] was used for FTC determination. A mixture of 4 mg of sample (final concentration of 0.02% w/v) in 4 ml of 99.5% ethanol, 4.1 ml of 2.51% linoleic acid in 99.5% ethanol, 8.0 ml of 0.02 M phosphate buffer (pH 7.0) and 3.9 ml of distilled water contained in screw cap vial (Ø38 × 75 mm) was placed in an oven at 40°C in the dark. To measure the extent of antioxidant activity, 0.1 ml of the reaction mixture was transferred to a test tube (Ø38 × 150 mm) and, to it; 9.7 ml of 75% (v/v) aqueous ethanol, followed by 0.1 ml of 30% aqueous ammonium thiocyanate and 0.1 ml of 0.02 M ferrous chloride in 3.5% hydrochloric acid were added. Three minutes after the addition of ferrous chloride to the reaction mixture, the absorbance of the resulting mixture (red colour) was measured at 500 nm every 24 h until the absorbance of the control reached its maximum. Butylated hydroxyl toluene (BHT) (final concentration of 0.02% w/v) was used as positive control, while the mixture without the plant extract was used as the negative control.

### Thiobarbituric acid (TBA) method

The method of Ottolenghi [23] modified by Kikuzaki and Nakatani [24] was used for the determination of free radicals present in the aqueous leaf extract. The final sample concentration of 0.02% w/v from the same samples prepared for FTC assay was used. Two millilitres of 20% trichloroacetic acid and 2 ml of 0.67% of thiobarbituric acid were added to 1 ml of sample solution from the FTC method. The mixture was placed in a boiling water bath for 10 min and then centrifuged after cooling at 3000 rpm for 20 min. The absorbance activity of the supernatant was measured at 552 nm and recorded after it has reached its maximum.

### 2, 2-Diphenyl-1-Picrylhydrazyl (DPPH) assay

The method of Liyana-Pathiana and Shahidi [25] was used for the determination of scavenging activity of DPPH free radical. One ml of 0.135 mM DPPH prepared in methanol was mixed with 1.0 ml of aqueous extract ranging from 0.2-0.8 mg/ml. The reaction mixture was vortexed thoroughly and left in dark at room temperature for 30 min. The absorbance was measured spectrophotometrically at 517 nm. The scavenging ability of the plant extract was calculated using this equation;

where Abs_control _is the absorbance of DPPH + methanol; Abs_sample _is the absorbance of DPPH radical + sample (i.e. extract or standard).

### 2, 2'-azino-bis (3-ethylbenzthiazoline-6-sulphonic acid (ABTS) scavenging activity

The method of Re et al. [26] was adopted for the determination of ABTS activity of the plant extract. The working solution was prepared by mixing two stock solutions of 7 mM ABTS solution and 2.4 mM potassium persulphate solution in equal amount and allowed to react for 12 h at room temperature in the dark. The resulting solution was later diluted by mixing 1 ml of freshly prepared ABTS^.+ ^solution followed by the measurement of absorbance at 734 nm after 7 min. The percentage of scavenging inhibition capacity of ABTS^.+ ^of the extract was calculated and compared with Butylated hydroxyltoluene (BHT). The percent of scavenging inhibition capacity of ABTS^.+ ^of the extract was calculated from the following equation:

### Nitric oxide scavenging activity

The method of Garratt [27] was adopted to determine the nitric oxide radical scavenging activity of aqueous extract of *H. longifolium*. Sodium nitroprusside in aqueous solution at physiological pH spontaneously generate nitric oxide which interacts with oxygen to produce nitrite ions determined by the use of Griess reagents. Two millilitre of 10 mM sodium nitroprusside dissolved in 0.5 ml phosphate buffer saline (pH 7.4) was mixed with 0.5 ml of plant extract at various concentrations (0.2-0.8 mg/ml). The mixture was incubated at 25°C. After 150 min, 0.5 ml of incubation solution was withdrawn and mixed with 0.5 ml of Griess reagent [(1.0 ml sulfanilic acid reagent (0.33% in 20% glacial acetic acid at room temperature for 5 min with 1 ml of naphthylethylenediamine dichloride (0.1% w/v)]. The mixture was incubated at room temperature for 30 min. The absorbance was measured at 540 nm. The amount of nitric oxide radical was calculated following this equation:

Where A_0 _is the absorbance before reaction and A_1 _is the absorbance after reaction has taken place.

### Scavenging activity of superoxide anion

The scavenging activity of superoxide anion was determined by the method of Yen and Chen [28]. The reaction mixture consists of 1 ml of plant extract (1 mg/ml), 1 ml of PMS (60 μM) prepared in phosphate buffer (0.1 M pH 7.4) and 1 ml of NADH (phosphate buffer) was incubated at 25°C for 5 min, the absorbance was read at 560 nm against blank samples.

### Hydrogen peroxide scavenging activity

Scavenging activity of hydrogen peroxide by the plant extract was determined by the method of Ruch et al. [29]. Plant extract (4 ml) prepared in distilled water at various concentration was mixed with 0.6 ml of 4 mM H_2_O_2 _solution prepared in phosphate buffer (0.1 M pH 7.4) and incubated for 10 min. The absorbance of the solution was taken at 230 nm against blank solution containing the plant extract without H_2_O_2_.

## Results

### Phytochemical Screening

The phytochemical analysis conducted on *H. longifolium *extract revealed the presence of tannins, flavonoids, steroids and saponins. The total phenol content of the aqueous leaf extract was 0.499 mg gallic acid equivalent/g of extract power. The total flavonoid and proanthocyanidin contents of the plant were 0.705 and 0.005 mg gallic equivalent/g of extract powder respectively with reference to standard curve (Y = 0.0067x+0.0132, r^2 ^= 0.999) (Table 1). These phytochemical compounds are known to support bioactive activities in medicinal plants and thus responsible for the antioxidant activities of this plant extract used in this study.

**Table 1:** The phytochemical components of *H. longifolium *based on the preliminary aqueous crude leaf extract screening.

Phytochemical Compounds	Presence	Extract equivalent of Gallic (mg/g)
Tannins	+	ND
Flavonoids	++	ND
Steroids	+++	ND
Alkaloids	-	ND
Saponins	+	ND
Total phenol	+++	0.499
Total flavonoids	+++	0.705
Total proanthocyanidin	+	0.005

+++ = appreciable amount (positive within 5 mins.); ++ = moderate amount (positive after 5 mins. but within 10 mins); + = trace amount (positive after 10 mins. but within 15 mins); - = completely absent.

### Free radical scavenging activities

#### Total Antioxidant Capacity

**Figure 1:**
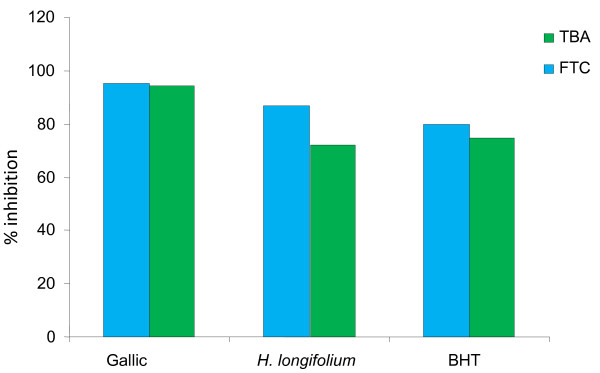
**Antioxidant properties of plant extract compared to the standards (Gallic and BHT) as determined with the FTC (500 nm) and TBA (552 nm) methods on the 6th day**. **TBA**: Thiobarbituric acid. **FTC**: Ferric thiocyanate. **BHT**: Butylated hydroxyl toluene.

The *in vitro *antioxidant assay of the plant extract (Figure 1) reveals significant antioxidant potential compared with standard BHT and gallic acid (final concentration of 0.02% w/v). The percentage inhibition of lipid peroxide at the initial stage of oxidation showed antioxidant activity of 87% compared to BHT (84.6%) and gallic acid (96%), and the percentage inhibition of malondialdehyde by the extract showed percentage inhibition of 78% compared to both BHT (72.24%) and gallic (94.82%).

#### The reducing power potentials of the extract

**Figure 2:**
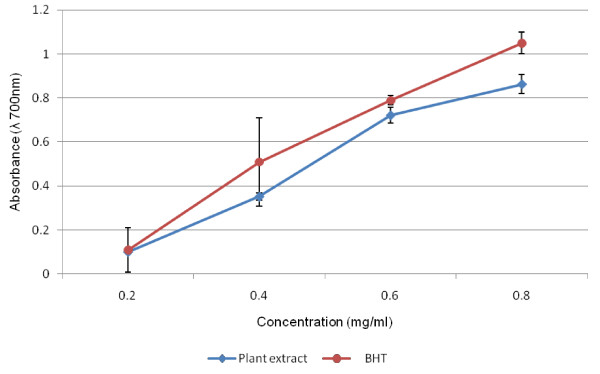
**Reducing power activities of the aqueous extract of *H. longifolium *in comparison with a standard (BHT) at λ = 700 nm**. **BHT**: Butylated hydroxyl toluene.

Figure 2 shows the reducing power potentials of the aqueous extract of the test plant in comparison with a standard BHT at 700 nm. The reducing capacity of the extract, another significant indicator of antioxidant activity was also found to be appreciable. In the reducing power assay, the presence of antioxidants in the sample would result in the reduction of Fe^3+ ^to Fe^2+ ^by donating an electron. The amount of Fe^2+ ^complex can then be monitored by measuring the formation of Perl's blue at 700 nm. Increasing absorbance indicates an increase in reductive ability. The results show that there was increase in reducing power of the plant extract as the extract concentration increases.

#### Scavenging activities inhibition by the extract

The percentage inhibition of scavenging activities of the aqueous extract of the test plant for DPPH, ABTS, hydrogen peroxide, nitric oxide and superoxide anion radical were shown in Table 2. The ABTS and nitric oxide radical scavenging activity of the extract at 0.8 mg/ml, which was the highest concentration of the extract tested, was 75.10% and 66.91%. The plant extracts showed appreciable free radical scavenging activities at the highest concentrations of 0.8 mg/ml on hydrogen peroxide, superoxide anion radical and DPPH. The percentage inhibitions are 72.44%, 75.91% and 64.96% for hydrogen peroxide, DPPH and superoxide anion radicals respectively (Table 2).

**Table 2:** Radical scavenging activities of aqueous crude leaf extract of *H. longifolium *and BHT as standard at different concentrations.

Percentage inhibition (% I) of radical scavenging of *H. longifolium*					
**Extract or BHT concentration** **(mg/ml)**	**Superoxide anion**	**Nitric oxide**	**DPPH**	**Hydrogen peroxide**	**ABTS**

0.2	62.65(60.16)	31.71(40.27)	40.91(42.62)	58.99(68.61)	50.00(51.17)
0.4	67.55(73.49)	42.00(46.27)	47.63(53.00)	66.14(73.29)	59.31(63.39)
06	72.16(77.12)	62.28(61.87)	58.33(73.99)	70.85(76.22)	66.31(77.20)
0.8	75.01(79.96)	64.96(80.29)	75.91(82.32)	72.44(80.00)	75.10(77.95)

BHT values in parenthesis.

## Discussion

The phytochemical analysis conducted on *H. longifolium *extract revealed the presence of tannins, flavonoids, steroids and saponins. Tannins are known to be useful in the treatment of inflamed or ulcerated tissues and they have remarkable activity in cancer prevention and anticancer [30,31]. Thus, *H. longifolium *containing this compound may serve as a potential source of bioactive compounds in the treatment of cancer.

Flavonoids have been shown to exhibit their actions through effects on membrane permeability, and by inhibition of membrane-bound enzymes such as the ATPase and phospholipase A2 [32], and this property may explain the mechanisms of antioxidative action of *H. longifolium*. Flavonoids serve as health promoting compound as a results of its anion radicals [33]. These observations support the usefulness of this plant in folklore remedies in the treatment of stress-related ailments and as dressings for wounds normally encountered in circumcision rites, bruises, cuts and sores [11,12,34,35].

Also, the plant extract was revealed to contain saponins, known to produce inhibitory effect on inflammation [36] and are major ingredients in traditional Chinese medicine and thus responsible for most of the observed biological effects [37], and this tend to justify the use of *H. longifolium *in traditional medicine. The plant extract was also positive for steroids which are very important compounds especially due to their relationship with compounds such as sex hormone [38]. The presence of these phenolic compounds in this plant contributed to their antioxidative properties and thus the usefulness of these plants in herbal medicament. Phenols have been found to be useful in the preparation of some antimicrobial compounds such as dettol and cresol. This plant is used routinely among many tribes in Africa for the treatment of various diseases.

Alkaloid was not detected in this study plant. Alkaloids have been associated with medicinal uses for centuries and one of their common biological properties is their cytotoxicity [39], and their absence in this plant tend to lower the risk of poisoning by the plant.

The result of DPPH scavenging activity assay in this study indicates that the plant was potently active. This suggests that the plant extract contain compounds that are capable of donating hydrogen to a free radical in order to remove odd electron which is responsible for radical's reactivity. The ability of this plant extract to scavenge DPPH could also reflect its ability to inhibit the formation of ABTS+. The scavenging activity of ABTS+ radical by the plant extract was found to be appreciable; this implies that the plant extract may be useful for treating radical-related pathological damage especially at higher concentration [40].

Superoxide anion radical is one of the strongest reactive oxygen species among the free radicals that are generated [28]. The scavenging activity of this radical by the plant extract compared favourably with the standard reagents such as gallic acid suggesting that the plant is also a potent scavenger of superoxide radical.

Hydrogen peroxide is an important reactive oxygen species because of its ability to penetrate biological membranes. However, it may be toxic if converted to hydroxyl radical in the cell [41]. Scavenging of H_2_O_2 _by the plant extracts may be attributed to their phenolics, which donate electron to H_2_O_2_, thus reducing it to water. The extract was capable of scavenging hydrogen peroxide in a concentration dependent manner.

Nitric oxide (NO) is a reactive free radical produced by phagocytes and endothelial cells, to yield more reactive species such as peroxynitrite which can be decomposed to form OH radical. The level of nitric oxide was significantly reduced in this study by the crude extract. Since NO plays a crucial role in the pathogenesis of inflammation [42], this may explain the use of *H. longifolium *for the treatment of inflammation and for wound healing.

Plants with antioxidant activities have been reported to possess free radical scavenging activity [43]. Free radicals are known as major contributors to several clinical disorders such as diabetes mellitus, cancer, liver diseases, renal failure and degenerative diseases as a result of deficient natural antioxidant defence mechanism [44].

## Conclusions

This study affirms the *in vitro *antioxidant potential of crude extract of the leaf of *Helichrysum longifolium*, with results comparable to those of the standard compounds such as gallic acid and butylated hydroxyl toluene (BHT). Further studies are needed to clarify the *in vivo *potential of this plant in the management of human diseases resulting from oxidative stress and this is a subject of investigation in our group.

## Competing interests

The authors declare that they have no competing interests.

## Authors' contributions

OAA carried out the study design, plant collection, experimental work, data collection and interpretation, literature search and manuscript preparation. AIO provided assistance in data interpretation, supervised the work, evaluated the data and corrected the manuscript for publication. Both authors read and approved the final manuscript.

## Pre-publication history

The pre-publication history for this paper can be accessed here:

http://www.biomedcentral.com/1472-6882/10/21/prepub
